# Variation in COVID-19 vaccination and adverse outcomes: a state of Georgia case study

**DOI:** 10.1186/s12889-025-24260-4

**Published:** 2025-09-25

**Authors:** Akane B. Fujimoto, Pinar Keskinocak, Dima Nazzal

**Affiliations:** 1 H. Milton Stewart School of Industrial and Systems Engineering, Georgia Institute of Technology, 755 Ferst Dr NW, Atlanta, GA 30332 USA; 2 Center for Health and Humanitarian Systems, Georgia Institute of Technology, Atlanta, Georgia United States

**Keywords:** COVID-19, Vaccine uptake, Health policy

## Abstract

**Background:**

Differences in COVID-19 vaccine coverage, deaths, and hospitalizations across racial and ethnic groups have been documented in the United States. Evaluating these patterns at a smaller geographical scale while accounting for differences in vaccination uptake across racial groups provides additional insights. In this study, we quantify adult COVID-19 vaccination, death, and hospitalization outcomes across racial/ethnic groups and county urban-rural classification in the state of Georgia.

**Methods:**

This cross-sectional study included adult COVID-19 vaccination (primary series, 1st booster, and 2nd booster), COVID-19-related deaths, and COVID-19-positive hospitalizations reported to the Georgia Department of Public Health through February 2023. We computed vaccination, death, and hospitalization rates for Hispanic, non-Hispanic (NH) Asian, NH Black, and NH White adults by county of residence urban-rural classification and vaccination status. Rate ratios (RRs) were calculated to evaluate differences in outcomes across racial/ethnic groups.

**Results:**

There were 14,386,650 COVID-19 vaccine doses administered, 40,711 COVID-19-related deaths, and 138,024 hospitalizations recorded during the study period. Differences by race/ethnicity varied by outcome and county urban-rural class. NH Black adults were at higher risk of being hospitalized with COVID-19 than NH White adults (RR 1.77, 95% CI, 1.75–1.79), even when stratified by vaccination status. NH Black adults were at higher risk of dying of COVID-19 than NH White adults (RR, 1.37, 95% CI, 1.34–1.39), except among those who received a booster dose (RR, 0.96, 95% CI, 0.86–1.07).

**Conclusions:**

NH Black adults experienced higher rates of adverse COVID-19-related outcomes compared to other racial/ethnic groups, even among the vaccinated. Stratifying outcomes by race/ethnicity, county urban-rural class, and vaccination status provided a better understanding of patterns across populations and geographies for targeted interventions. Public health agencies should focus on improving up-to-date vaccination coverage and removing barriers to access to care among communities that are underserved, particularly NH Black individuals.

**Supplementary Information:**

The online version contains supplementary material available at 10.1186/s12889-025-24260-4.

## Background

The COVID-19 pandemic has resulted in a substantial loss of life, health, and well-being. Even though people across the United States (US) and the world have been affected by the pandemic, racial and ethnic minority groups have been more adversely impacted [1–4]. Hispanic and non-Hispanic (NH) Black individuals, in particular, have experienced worse health outcomes throughout the pandemic [5–9].

Although these trends have been widely studied at the national level [10, 11], it is important to evaluate trends at a smaller scale [12–17]. Virus exposure, vaccine supply and hesitancy, and access to treatment significantly varied geographically, as state and local public health departments implemented plans of action [18]. In this study, we focus on analyzing COVID-19 outcomes in Georgia. Georgia is the 8th most populous state in the US. It has the highest NH Black population proportion and is the third most diverse among the top 10 most populous states (Table 1 in the Supplement).

Observations from this analysis can uncover trends that are otherwise masked when looking at more aggregated data. In addition, this analysis highlights the importance of data collection for adequate public health efforts, especially regarding breakthrough deaths and hospitalizations, as these datasets are more limited. For example, while the Centers for Disease Control and Prevention (CDC) reports COVID-19 vaccination, case, and death rates by race/ethnicity at the national and state level, it does not currently report breakthrough outcomes by race/ethnicity [19]. The analysis of outcomes by vaccination status accounts for differences in vaccination coverage across racial groups, which can lead to more effective policies [20, 21]. To our knowledge, most published studies on racial/ethnic differences in COVID-19 health outcomes have not analyzed outcomes by vaccination status, in part due to data reporting limitations.

The objective of this paper is to quantify differences in adult COVID-19 vaccination, death, and hospitalization outcomes by race/ethnicity and county of residence urban-rural classification in Georgia.

## Methods

### Study design

This cross-sectional study included COVID-19 vaccination, COVID-19-related deaths, and COVID-19-positive hospitalizations reported to the Georgia Department of Public Health (GDPH) from the start of data collection (March 3, 2020, for deaths and hospitalizations and December 14, 2020, for vaccination) to February 28, 2023. This study included records of individuals aged 18 years or older who reported Georgia as their state of residence.

### Data sources

GDPH collected individual-level records from multiple sources, including electronic laboratory reports (ELRs) submitted by laboratories, hospitals, and healthcare providers. ELRs included patient identifiers, test information, and results. In addition to ELRs, providers and other required reporters submitted individual “case” reports [22].

When new records were received, they were automatically matched and merged with existing records for the same individual, when applicable. GDPH also used additional data sources, such as death certificates, voter registration records, and driver services data, to complete missing demographics and residential information. Before releasing the data for analysis, GDPH removed all personally identifiable information, including names, full addresses, phone numbers, and dates of birth. More detailed information about GDPH’s data collection and processing procedures is available online [22].

The resulting de-identified dataset included individual-level records of COVID-19 vaccination, hospitalization, and death. Each record contained a unique anonymized person ID, along with age, race/ethnicity, county of residence, and the date and county where the vaccination, hospitalization, or death occurred. For hospitalization and death records, the individual’s vaccination status was also included when available.

### Definitions

Vaccination records were included in the analysis if the individual was a Georgia resident and at least 18 years old at the time of their latest vaccination dose. If the individual was 18 years old at the time of their 1st booster, but under 18 when they received their primary series, their primary series record is included in the analysis.

The study included records of Georgia residents; however, a significant number of vaccination records had missing residence information (county or state). Since the inclusion/exclusion of these records influences rate calculations, we calculated both unimputed and imputed vaccination rates. In the unimputed analysis, records missing residence information were excluded. In the imputed analysis, we addressed missing county of residence by assuming that individuals resided in the same county where they received their vaccine. For example, if a record lacked residence information but indicated the vaccination occurred in Fulton County, we imputed the individual’s residence as Fulton County. The county of vaccine administration was consistently reported in the dataset.

Individuals were classified as fully vaccinated if they received an FDA-authorized or approved primary series. The vaccine dose number was not explicitly recorded after individuals received their primary series. Therefore, GDPH labeled additional doses as boosters using the individual’s unique ID, date of vaccination, and time from the last vaccination dose. Individuals were classified as boosted with a 1st booster if they completed the primary series, received an additional dose on or after August 13, 2021, and it was administered at least 28 days after their primary series was completed. They were classified as boosted with a 2nd booster if they received an additional dose on or after April 29, 2022, and it was at least one day after their 1st booster. The dates used for the dose classification aligned with the release of the boosters.

COVID-19-related deaths were defined as confirmed (PCR test) or probable (antigen test) COVID-19 cases that were either reported as deceased by healthcare providers or medical examiners/coroners, identified by death certificates with COVID-19 indicated as the cause of death or with evidence that COVID-19 contributed to the individual’s death. GDPH followed the CDC/CSTE COVID-19 death definition [22, 23].

COVID-19-positive hospitalizations included confirmed and probable cases that were hospitalized for any reason at the time the case was reported or when the case was interviewed [22]. Hospitalizations that occurred after a case was reported were not captured. Therefore, the records included are likely an underestimation of the actual number of hospitalizations.

Deaths and hospitalizations were classified by the vaccination status of the individual at the time of the outcome, if available, which included unvaccinated or partially vaccinated (individuals who did not complete the primary series), vaccinated with a primary series only, and vaccinated with a primary series and at least one booster dose. Breakthrough outcomes included individuals who completed their primary series or booster dose 14 or more days before specimen collection and did not receive a positive COVID-19 laboratory test within 90 days before the current positive test [24]. Any references to “deaths” or “hospitalizations” will hereafter refer to the outcomes as defined above.

### Rate calculations and analysis

**Fig. 1:**
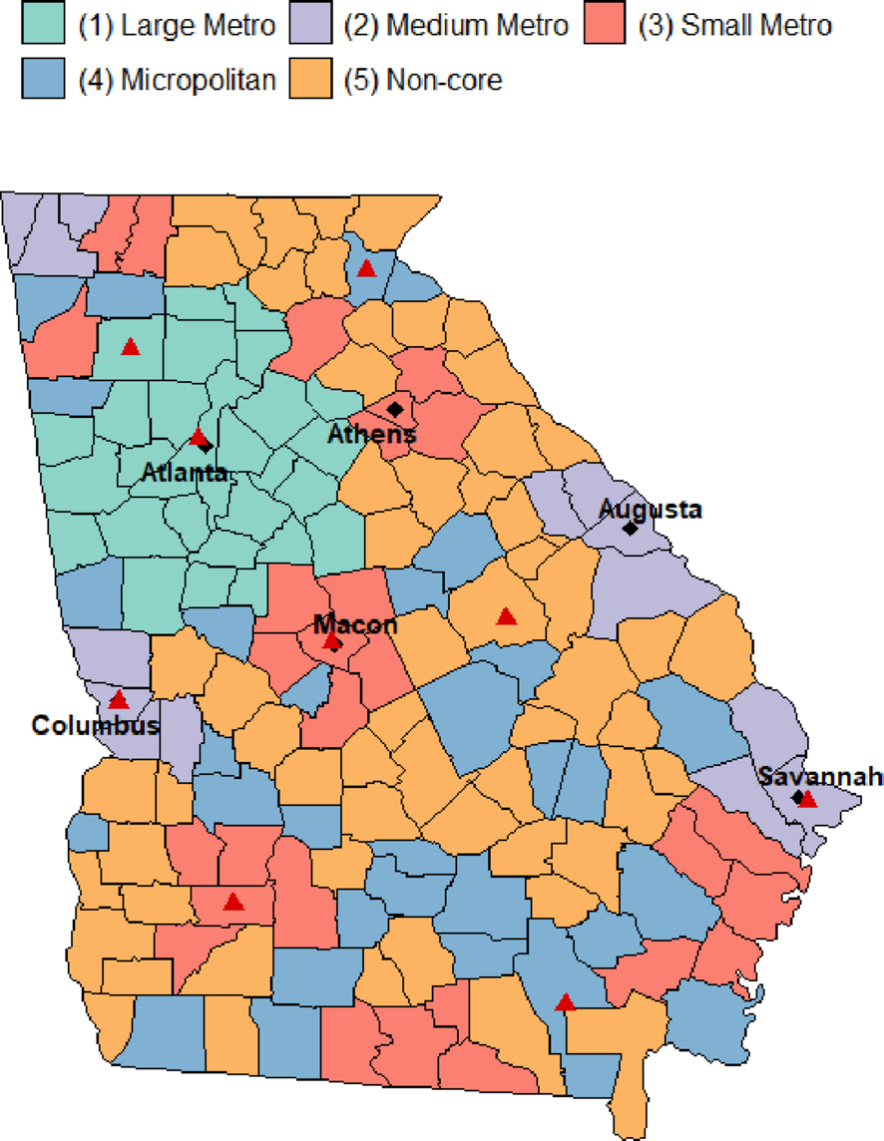
Map of Georgia counties by 2013 NCHS urban-rural classification. ◆ Large city; ▲ Mass vaccination site

Vaccination, death, and hospitalization counts were aggregated by race/ethnicity and urban-rural county classification using the 2013 NCHS county urban-rural classification scheme [25]. This scheme categorizes counties into six levels, from most urban to most rural: large central metro, large fringe metro, medium metro, small metro, micropolitan, and non-core. Counties were classified based on their designation as part of a metropolitan or micropolitan statistical area and their population size. For our analysis, we combined “large central metro” and “large fringe metro” into a single “large metro” class, as the former only contained a single county (Fig. 1). The race/ethnicity groups included Hispanic or Latino (of any race) (Hispanic), NH Asian, NH Black or African American (NH Black), and NH White. NH American Indian or Alaska Native and NH Native Hawaiian or Other Pacific Islander adults were not included in the race/ethnicity-specific analysis as their population makes up less than 0.5% of the adult population in Georgia [26], leading to estimates that are not as reliable for comparison (Table 2 in the Supplement).

Vaccination rates were calculated per 100 adults, while death and hospitalization rates were calculated per 100,000 adults, using 2019 population counts [26]. Breakthrough deaths and hospitalization rates were calculated using the unimputed vaccination counts from the dataset analyzed as the denominator. We computed age-adjusted rates for death and hospitalization outcomes to account for differences in the population age distribution by race/ethnicity and county class. Counts were stratified by age groups (18–29, 30– 39, 40– 49, 50– 59, 60– 69, 70– 79, and 80 + years), and the 2019 Georgia population was used as the standard population for age adjustment [26]. Specifically, we applied the proportion of the population in each age group from the 2019 Georgia population data as weights in the direct age standardization method [27]. Rates were compared across racial/ethnic and urban-rural classes.

Rate ratios (RRs) were calculated to assess relative differences by dividing the vaccination, death, and hospitalization rates among NH Asian, NH Black, and Hispanic adults by the corresponding rates among NH White adults. We used bootstrapping to calculate 95% confidence intervals (CIs) around the RRs (see Supplemental Materials for details on the calculations). A supplemental analysis was also performed to evaluate the variation in vaccination and outcome rates across counties. Data analysis was conducted using the software R [28].

## Results

A total of 14,386,650 vaccine doses were administered to Georgia adult residents since vaccination efforts started on December 14, 2020, through February 28, 2023, including primary series doses and 1st and 2nd boosters. A total of 40,711 COVID-19 deaths and 138,024 hospitalizations were reported from March 3, 2020, to February 28, 2023 (Table 1).

### Data missingness

6% of vaccination records had missing residence state and/or county. Residence missingness decreased in adults with additional doses (6% of primary series, 2.7% of 1st booster, and 1.5% of 2nd booster records were missing residence data). Hispanic adults had higher residence missingness (17.7%) than other races (3.7–7.4%) (Table 3 in the Supplement).

**Table 1:** Summary of vaccines administered to Georgia resident adults (18+) in Georgia from the beginning of vaccine distribution (December 14, 2020) to February 28, 2023, by vaccination dose and COVID-19-related deaths and COVID-19-positive hospitalizations among adults from the first event recorded (March 3, 2020) to February 28, 2023

		Vaccination				Adverse outcomes	
		All doses	Fully vaccinated	1st booster	2nd booster	COVID-19-related deaths	COVID-19- positive hospitalizations
Total		14,386,650	5,152,318	2,629,923	985,378	40,711	138,024
Race/ethnicity N (%)	Hispanic or Latino	902,507 (6.3%)	354,830 (6.9%)	142,291 (5.4%)	35,614 (3.6%)	1,808 (4.4%)	10,380 (7.5%)
	NH Asian	776,770 (5.4%)	269,704 (5.2%)	169,899 (6.5%)	52,896 (5.4%)	645 (1.6%)	2,126 (1.5%)
	NH Black or African American	3,728,509 (25.9%)	1,347,836 (26.2%)	666,698 (25.4%)	241,947 (24.6%)	12,866 (31.6%)	54,329 (39.4%)
	NH White	6,886,149 (47.9%)	2,442,289 (47.4%)	1,266,237 (48.1%)	505,174 (51.3%)	25,113 (61.7%)	67,447 (48.9%)
	NH American Indian or Alaska Native	36,231 (0.3%)	12,784 (0.2%)	6,922 (0.3%)	2,405 (0.2%)	35 (0.1%)	86 (0.1%)
	NH Native Hawaiian or Other Pacific Islander	24,837 (0.2%)	9,393 (0.2%)	3,676 (0.1%)	1,188 (0.1%)	21 (0.1%)	98 (0.1%)
	NH Other	1,786,253 (12.4%)	614,224 (11.9%)	348,706 (13.3%)	141,034 (14.3%)	129 (0.3%)	1,845 (1.3%)
	Not reported	245,394 (1.7%)	101,258 (2%)	25,494 (1%)	5,120 (0.5%)	94 (0.2%)	1,713 (1.2%)
Age	Mean (Standard Deviation)	52.5 (18.3)	49.9 (18.4)	55.5 (17.9)	62.2 (15.6)	71.7 (14.7)	59.6 (18.7)
	Not Reported (N (%))	–	–	–	–	–	236 (0.2%)

*NH* Non-Hispanic

### Vaccination rate

#### Statewide

**Fig. 2:**
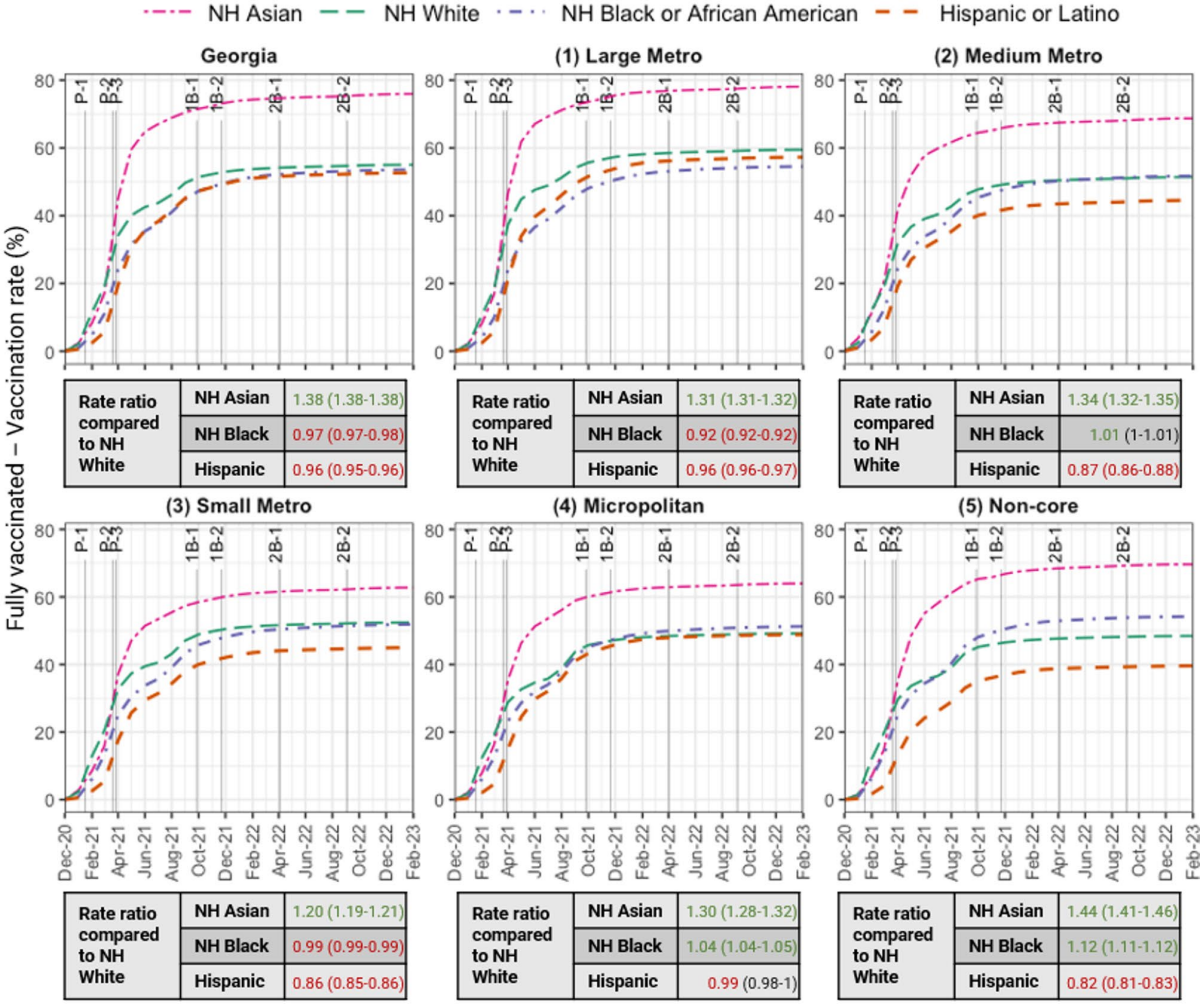
Monthly cumulative fully vaccinated rate by county urban-rural classification, stratified by race/ethnicity through February 28, 2023. Key vaccine rollout and booster guideline dates are annotated (see footnote 1). *NH* Non-Hispanic 1. Important dates are labeled as follows: Primary series vaccine rollout – P-1: Adults 65+ and first responders (January 2021), P-2: Adults 55+ and 16+ with medical conditions (March 2021), P-3: Individuals 16+ (March 2021); 1st booster guidelines – 1B-1: High-risk populations (September 2021), 1B-2: Adults 18+ (November 2021); 2nd booster recommendations – 2B-1: Older adults and immunocompromised (April 2022), 2B-2: Updated, bivalent booster for individuals 12+ (September 2022) 2. County of residence was not imputed and rate ratios (95% confidence intervals) of the vaccination rates among NH Asian, NH Black, and Hispanic adults compared to NH White adults are displayed

**Fig. 3:**
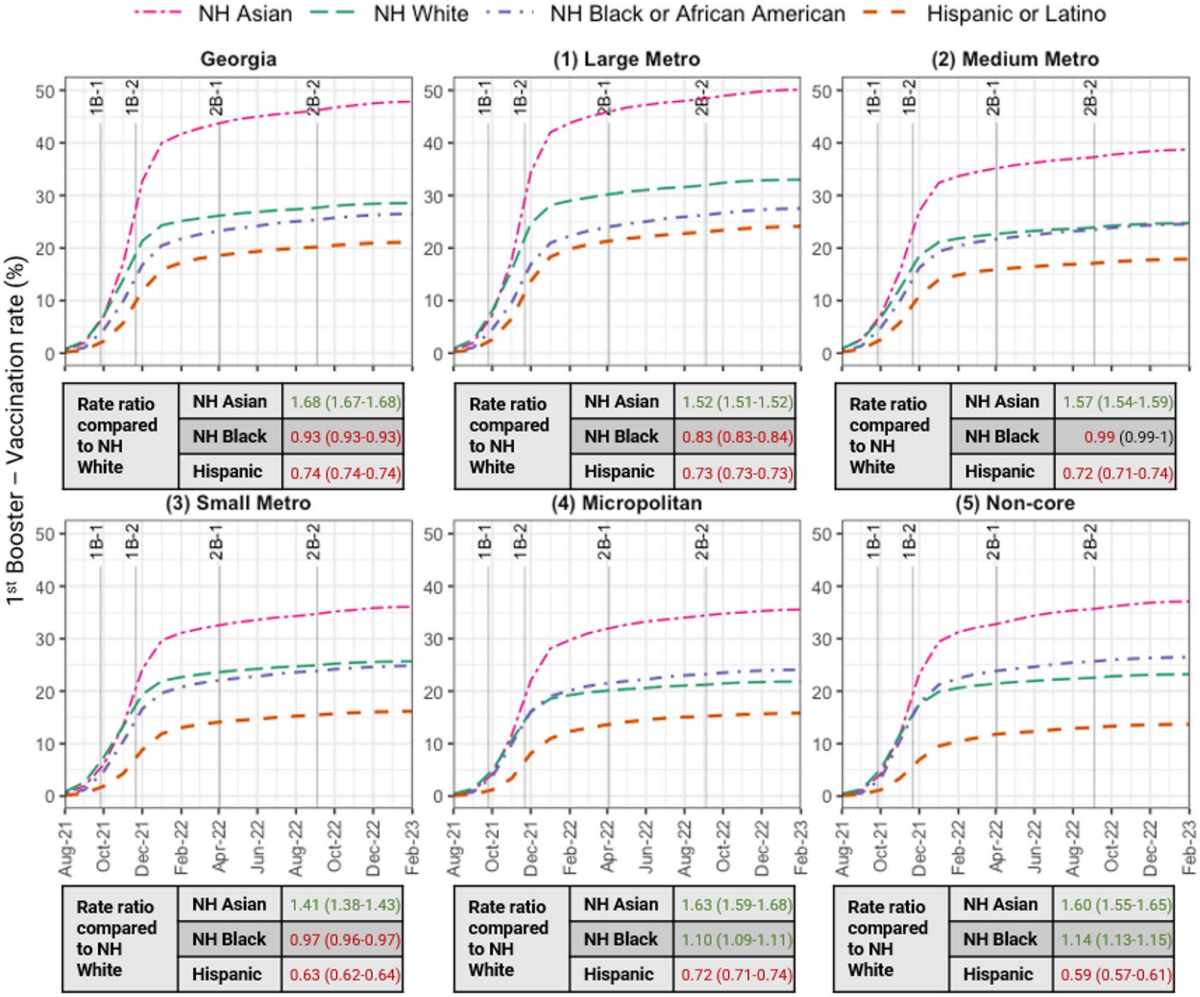
Monthly cumulative 1st booster vaccination rate by county urban-rural classification, stratified by race/ethnicity through February 28, 2023. Key booster guideline dates are annotated (see footnote 1) Important dates are labeled as follows: 1st booster guidelines – 1B-1: High-risk populations (September 2021), 1B-2: Adults 18+ (November 2021); 2nd booster recommendations – 2B-1: Older adults and immunocompromised (April 2022), 2B-2: Updated, bivalent booster for individuals 12+ (September 2022). *NH* Non-Hispanic 1. Important dates are labeled as follows: 1st booster guidelines – 1B-1: High-risk populations (September 2021), 1B-2: Adults 18+ (November 2021); 2nd booster recommendations – 2B-1: Older adults and immunocompromised (April 2022), 2B-2: Updated, bivalent booster for individuals 12+ (September 2022) 2. County of residence was not imputed and rate ratios (95% confidence intervals) of the vaccination rates among NH Asian, NH Black, and Hispanic adults compared to NH White adults are displayed

Cumulative primary series (Fig. 2), 1st booster (Fig. 3), and 2nd booster (Fig. 1 in the Supplement) vaccination rates (not imputed) were 75.9%, 47.8%, and 14.9% for NH Asian adults; 53.6%, 26.5%, and 9.6% for NH Black adults; 55%, 28.5%, and 11.4% for NH White adults; and 52.6%, 21.1%, and 5.3% for Hispanic adults, respectively. Refer to Fig. 2 in the Supplement for imputed vaccination rates.

NH Black adults had lower vaccination rates compared to NH White adults, primary series RR 0.97 (CI: 0.97–0.98); 1st booster RR 0.93 (CI: 0.93–0.93), and 2nd booster RR 0.85 (CI: 0.84–0.85). Hispanic adults had the lowest booster rates in the state, 21.1% compared to 26.5–47.8% in the other groups for the 1st booster and 5.3% compared to 9.6–14.9% for the 2nd booster. County of residence missingness only significantly affected primary series vaccination rates for Hispanic adults. When residence information was excluded, Hispanic adults had the lowest primary vaccination rates (52.6%). However, when these records were included, Hispanic rates increased to 64%.

#### County class

Vaccination rates for the primary series decreased with decreasing urbanization. Rates were 68.5% in the large metro county class, followed by 59.4% in the medium metro, 58.5% in the small metro, 54.9% in the micropolitan, and 54.4% in the non-core county classes. Similarly, 1st booster vaccination rates declined from 36.7% in the large metro county class to 28.6%, 28.1%, 24.5%, and 26% in the medium metro, small metro, micropolitan, and non-core county classes, respectively. For the 2nd booster, the rates were 14.1%, 10.5%, 10.2%, 8.2%, and 9.4% across the county classes (Table 4 in Supplement).

In the large metro county class, NH Black adults had a lower primary series vaccination rate compared to NH White adults (RR 0.92, CI: 0.92–0.92); 1st booster (RR 0.83, CI: 0.83–0.84), and 2nd booster (RR 0.73, CI: 0.73–0.74) rates. Similarly, NH Black adults had similar or lower vaccination coverage than NH White adults in the medium and small metro county classes. However, the NH Black vaccination rate was higher than the NH White rate in rural counties: micropolitan county class (RR 1.04, CI: 1.04–1.05; RR 1.10, CI: 1.09–1.11; and RR 1.10, CI: 1.09–1.12 for primary series, 1st booster, and 2nd booster, respectively) and non-core county class (RR 1.12, CI: 1.11–1.12; RR 1.14, CI: 1.13–1.15; and RR 1.08, CI: 1.06–1.09 for primary series, 1st booster, and 2nd booster, respectively) (Table 4 in the Supplement).

Hispanic adults had the lowest booster rates (13.7–24% for the 1st booster and 2.8–6.3% for the 2nd booster) in every county class. NH Asian adults had the highest vaccination rate (62.8–78.1%, 35.6–50.1%, and 8.7–15.8% for the primary series, 1st booster, and 2nd booster, respectively) in every vaccination status and county class.

### Death and hospitalization rates

#### Statewide

The cumulative age-adjusted death rates per 100,000 population were 264.8, 630.1, 460.6, and 520.8 among NH Asian, NH Black, NH White, and Hispanic adults, respectively. Cumulative age-adjusted hospitalization rates per 100,000 population were 743.3, 2,368.6, 1,337.4, and 2,036.7 among NH Asian, NH Black, NH White, and Hispanic adults, respectively.

**Fig. 4:**
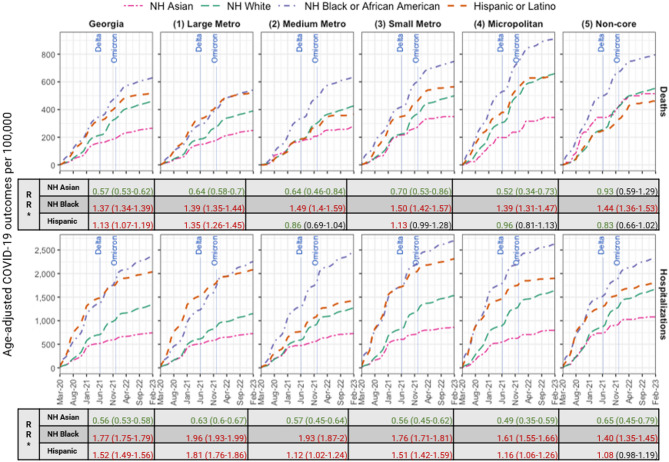
Monthly cumulative age-adjusted COVID-19-related deaths (top) and COVID-19-positive hospitalizations (bottom) per 100,000 adults by county urban-rural classification, stratified by race/ethnicity through February 28, 2023. The labels indicate when the Delta and Omicron variants became dominant. *NH* Non-Hispanic, *RR* Rate Ratio *Rate ratios (95% confidence intervals) of death and hospitalization rates among NH Asian, NH Black, and Hispanic adults compared to NH White adults are displayed

NH Black adults had the highest age-adjusted death and hospitalization rates in Georgia. Compared to NH White adults, NH Black adults had 1.37 (CI: 1.34–1.39) and 1.77 (CI: 1.75–1.79) times the risk of dying from COVID-19 or being hospitalized with COVID-19, respectively (Fig. 4). Hispanic adults had higher hospitalization rates than NH Black adults earlier in the pandemic before the Delta wave in the summer of 2021 (Fig. 4).

**Fig. 5:**
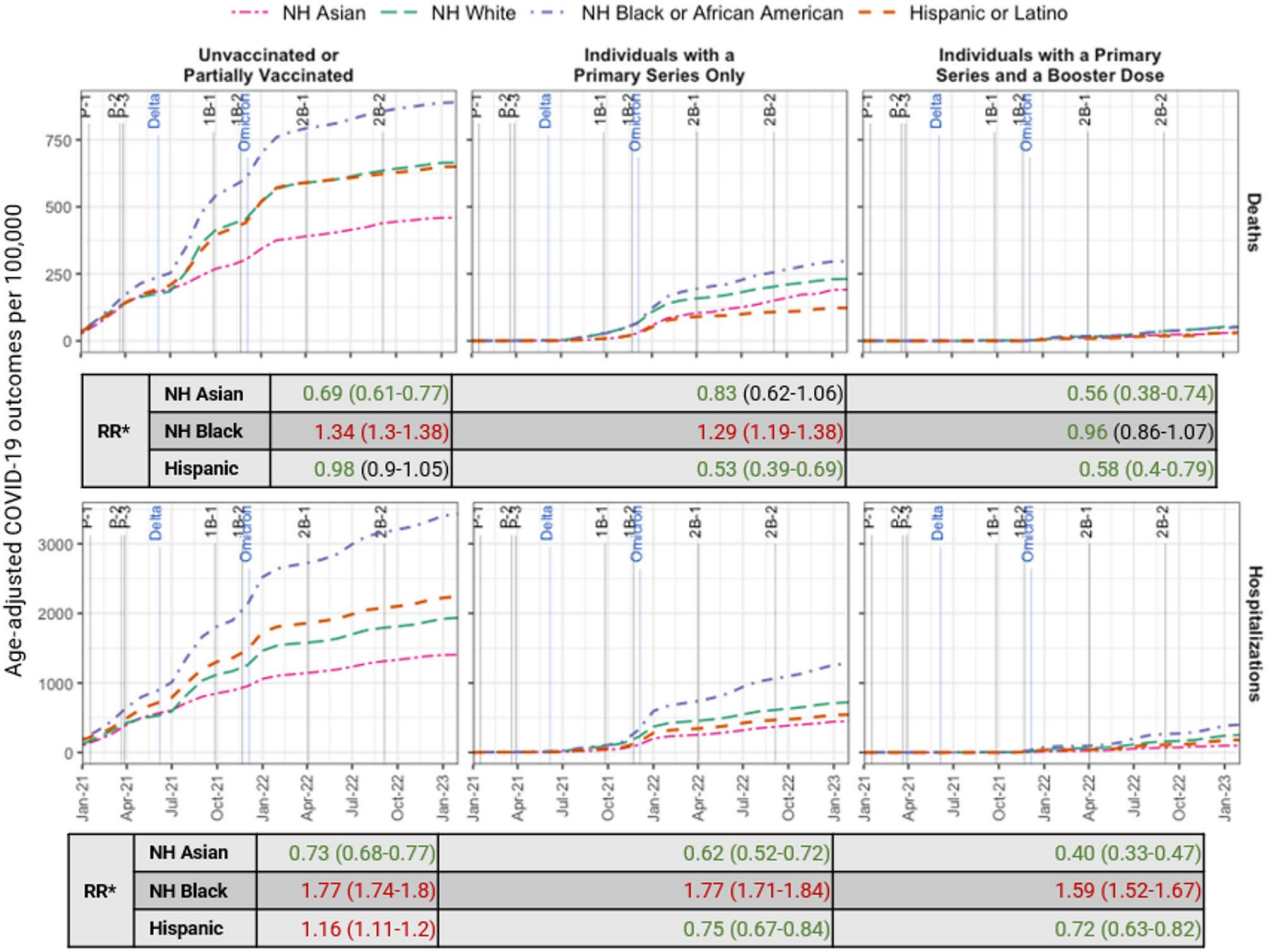
Monthly cumulative age-adjusted COVID-19-related deaths (top) and COVID-19-positive hospitalizations per 100,000 adults (bottom) by vaccination status and race/ethnicity in Georgia through February 28, 2023. Key vaccine rollout and booster guideline dates are annotated (see footnote 1), and blue labels indicate when variants became dominant. *NH* Non-Hispanic, *RR* Rate Ratio *Rate ratios (95% confidence intervals) of death and hospitalization rates among NH Asian, NH Black, and Hispanic adults compared to NH White adults are displayed 1. Important dates are labeled as follows: Primary series vaccine rollout – P-1: Adults 65+ and first responders (January 2021), P-2: Adults 55+ and 16+ with medical conditions (March 2021), P-3: Individuals 16+ (March 2021); 1st booster guidelines – 1B-1: High-risk populations (September 2021), 1B-2: Adults 18+ (November 2021); 2nd booster recommendations – 2B-1: Older adults and immunocompromised (April 2022), 2B-2: Updated, bivalent booster for individuals 12+ (September 2022) 2. County of residence was not imputed in the vaccination population counts used in the calculations

NH Black adults had the highest age-adjusted death rates per 100,000 population among unvaccinated or partially vaccinated individuals (889.7) and those who received a primary series only (297.6) (Fig. 5). Unvaccinated or partially vaccinated NH Black adults had 1.34 (CI: 1.3–1.38) times the risk of dying from COVID-19-related causes than their NH White counterparts. NH Black adults who received only the primary series had 1.29 (CI: 1.19–1.38) times the risk of death compared to their NH White counterparts. NH Black adults did not have a higher death risk among individuals with a primary series and a booster dose (RR 0.96, CI: 0.86–1.07).

NH Black adults had the highest age-adjusted hospitalization rates in all vaccination statuses, compared to NH White adults (RR 1.77 (CI: 1.74–1.8), 1.77 (CI: 1.71–1.84), and 1.59 (CI: 1.52–1.67)) among the unvaccinated or partially vaccinated, vaccinated with a primary series only, and vaccinated with a primary series and a booster dose, respectively (Fig. 5).

#### County class

Age-adjusted death rates increased with decreasing urbanization, with rates of 435 per 100,000 population in the large metro county class, followed by 489 in the medium metro, 555 in the small metro, 712 in the micropolitan, and 596 in the non-core county classes. We observed a similar trend in age-adjusted hospitalizations with rates of 1,582, 1,670, 1,897, 1,937, and 1,850 per 100,000 population in the large metro, medium metro, small metro, micropolitan, and non-core county classes, respectively (Table 5 in the Supplement).

Like statewide results, NH Black adults had the highest age-adjusted death rates across all county classes. Compared to NH White adults, NH Black adults had 1.39–1.5 and 1.4–1.96 times the risk of dying from COVID-19 or being hospitalized with COVID-19, respectively, depending on the county class (Fig. 4). Unvaccinated or partially vaccinated NH Black adults had a higher risk of dying from COVID-19 than their NH White counterparts in every county class (RR 1.29–1.60). Fully vaccinated NH Black adults had a higher risk of dying from COVID-19 across all county classes (RR 1.14–1.62), except in the non-core county class (RR 1.02, CI: 0.76–1.32). However, boosted NH Black adults did not have a higher mortality risk compared to NH White adults. Unvaccinated or partially vaccinated, fully vaccinated, and boosted NH Black adults had a higher risk of being hospitalized than their NH White counterparts across all county classes (RR 1.53–2.1), except among those who received a booster dose in the non-core county class (RR 1.03, CI: 0.85–1.22) (Tables 6 and 7 in the Supplement). A county-by-county comparison of vaccination, death, and hospitalization rates for NH Black and Hispanic adults versus NH White adults is provided in Appendix B.

## Discussion

The analysis revealed differences in outcomes by race/ethnicity that varied across county urban-rural classifications. Stratifying rates by race/ethnicity and county class uncovered patterns that would otherwise be masked when looking at state-wide results, which are largely driven by trends in the large metro counties.

### Outcomes among NH Black adults

NH Black vaccination rates were lower than NH White rates in Georgia. Differences in vaccination rates between NH White and NH Black adults, and between NH White and Hispanic adults, narrowed over time, similar to trends observed in the US [11, 29–31]. Vaccination uptake varied by county class, as observed in other states [32, 33]. NH Black adults’ primary series vaccination, 1st booster, and 2nd booster rates were lower than those of NH White adults in the large metro county class. In the micropolitan and non-core county classes, NH Black adults had higher vaccination coverage than NH White adults. NH White vaccination rates significantly decreased in rural counties, while NH Black vaccination rates stayed relatively stable compared to urban counties. This might indicate that vaccine hesitancy related to rural residency status more heavily impacts the NH White group than other racial groups. Previous research has shown that rural White adults were significantly less willing to be vaccinated than their non-rural counterparts. In contrast, rural and non-rural Black adults were equally as likely to be vaccinated [34]. Lower rural vaccination rates are associated with various factors, including lower educational attainment, fewer physicians per capita, and differences across county types, with lower rates observed in mining- and farming-dependent counties [35].

NH Black adults had the highest age-adjusted death and hospitalization rates across all county classes, as observed in the US [10]. When stratifying adverse outcomes by vaccination status, NH Black adults still had the highest age-adjusted death rates among the unvaccinated or partially vaccinated and the fully vaccinated. NH Black adults did not have the highest age-adjusted death rates among those who received a booster, which might suggest that staying up to date on vaccines with booster doses could help decrease differences in outcomes. Vaccinated NH Black adults had higher hospitalization rates than their counterparts in other racial groups. While widespread vaccination efforts can help reduce differences in COVID-19-related outcomes [11], our analysis shows that NH Black adults continue to experience worse outcomes than other groups, even among the fully vaccinated.

A surveillance-based study covering approximately 10% of the US population across 14 states found no statistical differences in ICU admission or mortality between NH White and NH Black adults within the unvaccinated, vaccinated with primary series, and boosted groups [36]. Research on breakthrough infections and illness has primarily focused on cohorts of patients with specific conditions, such as HIV, cancer, and hematologic malignancies [37–39]. Population-level studies on severe outcomes among the vaccinated remain limited in their ability to assess differences after adjusting for vaccination status, as data are often reported without sufficient granularity, particularly regarding race/ethnicity and age [20].

Gaps in health outcomes affecting the Black population are longstanding [40] and the COVID-19 pandemic has further amplified these challenges, regardless of vaccination status [41]. Some of the studied reasons for these gaps are rooted in structural barriers that perpetuate unequal access to timely and high-quality health services [42–47]. For example, in 2023, only 52% of Black individuals under age 65 had employer-sponsored or private health insurance, compared to 74% of White individuals [48].

Differences in health outcomes also stem from the unequal distribution of risk across racial groups [44, 49]. Black individuals have a higher prevalence of comorbidities, such as cardiovascular disease, which elevates the risk of severe outcomes from COVID-19 infection [50]. Additionally, minorities, including Black individuals, are at greater risk of exposure due to a higher likelihood of living in densely populated, multigenerational households and holding jobs in high-risk environments, where remote work and time off are limited [44, 51–54]. These high-risk conditions, coupled with limited access to quality healthcare and treatment, put minorities at an elevated risk of experiencing poorer health outcomes [43–47, 50, 52–54].

### Outcomes and data missingness among Hispanic adults

Primary series vaccination estimates for Hispanic adults were highly influenced by missing data. When records with missing residence information were excluded, Hispanic adults had the lowest vaccination rate in Georgia. On the other hand, when these records were included, Hispanic adults had the second highest vaccination rate, only behind NH Asian adults. Regardless of the imputation approach for residence information, Hispanic adults had better primary vaccination coverage than NH Black adults in the large metro county class.

Higher residence data missingness among Hispanic adults may be partly attributed to greater levels of medical mistrust within the Hispanic population [55], as well as factors related to migration and citizenship status that may hinder the disclosure of residence information [56]. Additionally, this missingness might be explained by the large number of Hispanic individuals employed as temporary agricultural workers or those who experience language barriers [57, 58].

Vaccination uptake among Hispanic adults lagged behind other groups during the initial vaccine rollout. However, the gap narrowed over time as vaccines became more accessible due to universal eligibility. This initial gap in vaccination may have contributed to higher rates of deaths and hospitalizations among Hispanic adults, as they had the highest rates before mid-2021.

The Hispanic 1st and 2nd booster vaccination rates were the lowest in Georgia and across all county classes. A large gap in booster uptake compared to the other groups, especially considering the decrease in the racial gap in the primary series, could be driven by the lack of targeted vaccination campaigns seen in the earlier phases, such as mass vaccination clinics and pop-up sites [59–61]. Booster vaccinations are more likely to occur in traditional settings like pharmacies and primary care settings, which are traditionally less accessible to minorities [62–64]. Hispanic adults also had the second-highest age-adjusted death and hospitalization rates in Georgia. Hispanic populations face many of the same barriers to healthcare access as NH Black communities [65], in addition to other factors such as language, migration, and citizenship status-related barriers [66–68].

### Outcomes for NH Asian adults

Finally, NH Asian adults consistently had higher vaccination coverage and lower death and hospitalization rates, regardless of county classification, as observed across the US [10]. The only exception was in the non-core county class, where NH Asian adults had the third-highest age-adjusted death rate. This elevated rate was driven by a surge in NH Asian deaths before vaccines became available. However, an analysis of deaths occurring after December 2020 showed that NH Asian adults, along with Hispanic adults, had the lowest age-adjusted death rates in the non-core county class.

### Limitations

The analysis used a dataset of all adult COVID-19 vaccinations, deaths, and hospitalizations reported to GDPH. However, there are limitations to the study.

First, several primary series vaccine records were missing residence information (6% of records). Assumptions about whether to classify these individuals as Georgia residents (as opposed to out-of-state residents) affect the calculation of fully vaccinated rates, especially within the Hispanic population, as they had a higher proportion of records with missing residence data. As a result, uncertainty around fully vaccinated coverage was greatest among Hispanic adults. Additionally, booster doses administered to individuals who received their primary series out of state were not included in the booster rates calculations, as they were potentially marked as primary series doses.

Second, hospitalizations in the dataset included individuals hospitalized for any reason and tested positive for COVID-19 during their hospital stay; they were not necessarily hospitalized due to COVID-19 complications. While this metric serves as a good indicator of COVID-19 adverse outcomes, specifically recording those hospitalized due to COVID-19 would provide a more accurate measure of the disease burden. Furthermore, the dataset only captured COVID-19-positive hospitalizations at the time the case was reported or interviewed. As a result, hospitalizations that occurred after the case was initially reported or interviewed were not included. Due to this, the number of hospitalizations reported is likely to underestimate the actual number of hospitalizations.

Third, counties were aggregated by county urban-rural classification, which provided findings for county classes, but removed analysis at the individual county level. To address this, we included an additional analysis in the supplemental materials comparing outcomes across individual counties. The analysis documents the variation within each county class and shows that, while inter-county variability exists, especially for vaccination coverage, the patterns observed at the county class level are consistent at the individual county level.

Fourth, the analysis did not account for external factors such as social determinants of health. It is important to extend this analysis to consider findings at the county level, supplemented with additional data that allows for better interpretability of the results.

## Conclusions

This study revealed differences in COVID-19-related outcomes across racial/ethnic groups and geographic areas, offering insights to guide public health decision-making in Georgia, with findings that may be relevant to other states. COVID-19 vaccination coverage, deaths, and hospitalizations in Georgia varied by race/ethnicity and urban-rural county classification. NH Black adults were at higher risk of dying of COVID-19-related causes and being hospitalized with COVID-19 than other racial/ethnic groups overall and when stratified by vaccination status, except for deaths among those who received a booster.

As NH Black adults continue to experience worse health outcomes despite comparable vaccination uptake, public health efforts must address broader systemic issues that contribute to these gaps in health outcomes. Effective strategies should go beyond increasing vaccination coverage among minority groups and focus on ensuring access to preventive, diagnostic, and curative care. This includes expanding insurance coverage, improving physical access to healthcare facilities, and providing employment benefits such as paid medical leave. Additionally, addressing underlying risk factors, such as housing insecurity and unsafe working conditions, is critical to improving health outcomes in populations that are underserved.

## Supplementary Information

Below is the link to the electronic supplementary material.

Supplementary Material 1

## Data Availability

The data used in this study is available through the Public Health Information Portal data request process (https://dph.georgia.gov/phip-data-request). Part of the data presented is summarized in the following dashboards (https://chhs.gatech.edu/covid19-dashboard) [69].

## Abbreviations

NH: Non-Hispanic
RR: Rate ratio
CI: Confidence interval
GDPH: Georgia Department of Public Health
