# Supplementary material for: Variation in COVID-19 vaccination and adverse outcomes: a state of Georgia case study
Source: BMC Public Health. 2025 Sep 25;25:3128. doi: 10.1186/s12889-025-24260-4 (PMC12465598; doi:10.1186/s12889-025-24260-4)
Supplement: Supplementary file 1 — Supplementary Material 1 [file 12889_2025_24260_MOESM1_ESM.docx]

**Supplemental Content**

**Table 1**. Racial/ethnic distribution of the US population and the 10 most populous states from the American Community Survey (ACS) demographic and housing estimates (2022, 5-years estimates) [1].

**Table 2.** Percentage of Georgia’s 18+ population by county class and racial/ethnic groups using 2019 OASIS estimates [2].

**Appendix A.** Confidence interval (CI) calculation using bootstrapping.

**Figure A1:** Bootstrap sample (B = 1,000) for rate ratios (RRs) of non-Hispanic (NH) Asian and NH White fully vaccinated rates.

**Table 3**. Residence missingness among fully vaccinated records by race/ethnicity.

**Figure 1**: Monthly cumulative 2^nd^ booster vaccination rate (county of residence not imputed) by county urban-rural classification, stratified by race/ethnicity through February 28, 2023.

**Figure 2.** Not imputed (No IM) and imputed (IM) cumulative vaccination rates through February 28, 2023, by race/ethnicity in Georgia.

**Table 4.** Cumulative vaccination rates (county of residence not imputed) in Georgia and by county urban-rural classification through February 28, 2023.

**Table 5.** Cumulative crude and age-adjusted COVID-19-related deaths and COVID-19-positive hospitalizations in Georgia and by county urban-rural classification through February 28, 2023.

**Table 6.** Cumulative age-adjusted COVID-19-related deaths per 100,000 adults by vaccination status in Georgia and by county urban-rural classification through February 28, 2023.

**Table 7**. Cumulative age-adjusted COVID-19-positive hospitalizations per 100,000 adults by vaccination status in Georgia and by county urban-rural classification as of February 28, 2023.

**Appendix B.** County-level analysis

**Figure B1.** Vaccination (not imputed) and age-adjusted death and hospitalization rates for non-Hispanic (NH) Black and NH White adults by county.

**Figure B2**. Vaccination (not imputed) and age-adjusted death and hospitalization rates for Hispanic and non-Hispanic (NH) White adults by county.

**Table B1**. Mean vaccination, age-adjusted death, and hospitalization rate ratios across counties, grouped by county urban-rural classification.

**References.Table 1**. Racial/ethnic distribution of the US population and the 10 most populous states from the American Community Survey (ACS) demographic and housing estimates (2022, 5-years estimates) [1].

|  | Total 18+ Population | % of the US 18+ Population | NH Asian (%) | NH Black  or African American (%) | NH White (%) | Hispanic or Latino (%) | NH American Indian or Alaska Native (%) | NH Native Hawaiian or Other Pacific Islander (%) |
| --- | --- | --- | --- | --- | --- | --- | --- | --- |
| US | 257,883,888 | - | 5.7 | 12.1 | 58.9 | 18.7 | 0.6 | 0.17 |
| (1) California | 30,581,534 | 11.9 | 14.9 | 5.3 | 35.2 | 39.7 | 0.3 | 0.34 |
| (2) Texas | 21,847,214 | 8.5 | 5.1 | 11.8 | 40.1 | 39.9 | 0.2 | 0.08 |
| (3) Florida | 17,399,100 | 6.7 | 2.8 | 14.9 | 52.0 | 26.5 | 0.1 | 0.05 |
| (4) New York | 15,865,936 | 6.2 | 8.8 | 13.8 | 53.8 | 19.5 | 0.2 | 0.03 |
| (5) Pennsylvania | 10,324,646 | 4.0 | 3.6 | 10.4 | 74.5 | 8.1 | 0.1 | 0.02 |
| (6) Illinois | 9,937,157 | 3.9 | 5.7 | 13.7 | 59.6 | 17.8 | 0.1 | 0.02 |
| (7) Ohio | 9,181,361 | 3.6 | 2.4 | 12.2 | 77.2 | 4.2 | 0.1 | 0.03 |
| (8) Georgia | 8,209,780 | 3.2 | 4.3 | 31.1 | 50.8 | 10.1 | 0.1 | 0.05 |
| (9) North Carolina | 8,186,326 | 3.2 | 3.1 | 20.6 | 61.7 | 10.0 | 0.9 | 0.06 |
| (10) Michigan | 7,908,457 | 3.1 | 3.2 | 13.4 | 73.5 | 5.5 | 0.3 | 0.02 |

NH: Non-Hispanic

**Table 2.** Percentage of Georgia’s 18+ population by county class and racial/ethnic groups using 2019 OASIS estimates [2].

| County  Urban-Rural Classification | Number of Counties | % of Georgia 18+ Population | Demographic Makeup (%) | | | | | |
| --- | --- | --- | --- | --- | --- | --- | --- | --- |
|  |  |  | **NH Asian** | **NH Black**  **or African American** | **NH White** | **Hispanic or Latino** | **NH American Indian or Alaska Native** | **NH Native Hawaiian or Other Pacific Islander** |
| (1) Large Metro | 28 | 56.12 | 3.6 | 19.1 | 27.3 | 5.2 | 0.1 | 0.03 |
| (2) Medium Metro | 14 | 11.36 | 0.3 | 3.7 | 6.6 | 0.6 | 0.04 | 0.02 |
| (3) Small Metro | 28 | 14.57 | 0.3 | 3.8 | 8.8 | 1.4 | 0.04 | 0.01 |
| (4) Micropolitan | 31 | 9.29 | 0.1 | 2.4 | 6.0 | 0.6 | 0.03 | 0.01 |
| (5) Non-core | 58 | 8.67 | 0.1 | 2.0 | 6.1 | 0.5 | 0.03 | 0.00 |
| Georgia | 159 | - | 4.4 | 31.0 | 54.7 | 8.3 | 0.2 | 0.06 |

NH: Non-Hispanic

**Appendix A.** Rate ratio (RR) confidence interval (CI) calculation using bootstrapping.

We calculated the 95% confidence intervals (CIs) around the rate ratio (RR) using bootstrapping. We followed these steps [3]:

1. Define the RR:

$$RR= \frac{Rate in group A}{Rate in group B}=\frac{\frac{X_{A}}{N_{A}}}{\frac{X_{B}}{N_{B}}}$$

$$where X_{i}:Number of outcomes in group i$$

$$N_{i}:Total population in group i$$

2. Resample the data:
3. We create a dataset for each group where 1 indicates “had outcome” and 0 indicates “did not have outcome”.

Group A: 1, 1, 1, …0, 0 ,0 … ($X_{A}$ 1’s and ${N_{A}- X}_{A}$ 0’s)

Group B: 1, 1, 1, …0, 0 ,0 … ($X_{B}$ 1’s and ${N_{B}- X}_{B}$ 0’s)

1. We take a random sample with replacement for each group. The sample size is the same as the original for each group (i.e., $N_{A}$ and $N_{B}$).
2. Using the new samples, calculate the RR.
3. Repeat step 2b and 2c B times (e.g., B = 1,000).
4. Compute the CI

We calculate the 95% CI for the RR using the 2.5^th^ and 97.5^th^ percentiles from the bootstrap sample distribution.

Example:

We calculate the 95% CI for the fully vaccinated RR between NH Asian and NH White in non-core counties.

$$RR= \frac{FV Vax Rate NH Asian}{FV Vax Rate NH White}=\frac{69.64}{48.48}=1.44$$

The bootstrap sample with B =1,000 is shown in Figure A1. The confidence intervals using the 2.5^th^ and 97.5^th^ percentiles are 1.41 and 1.46. The RR (95% CI) is 1.44 (1.41, 1.46).


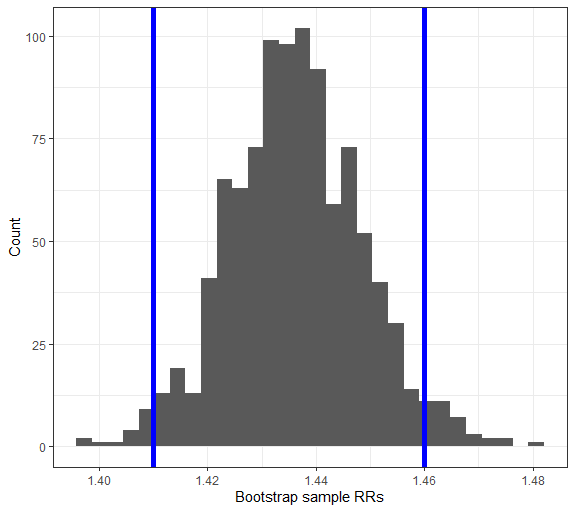


**Figure A1:** Bootstrap sample (B = 1,000) for rate ratios (RRs) of non-Hispanic (NH) Asian and NH White fully vaccinated rates.

**Table 3**. Residence missingness among fully vaccinated records by race/ethnicity.

| Race/Ethnicity | Total Records  (N) | Records Without  Residence Information  (N) | % Without Residence Information Within Group |
| --- | --- | --- | --- |
| Hispanic or Latino | 433,171 | 76,596 | 17.7 |
| NH Asian | 292,315 | 21,615 | 7.4 |
| NH Black or African American | 1,416,365 | 62,552 | 4.4 |
| NH White | 2,559,124 | 94,173 | 3.7 |
| NH American Indian or Alaska Native | 13,854 | 977 | 7.1 |
| NH Native Hawaiian or Other Pacific Islander | 10,045 | 548 | 5.5 |
| NH Other | 660,397 | 43,453 | 6.6 |
| Not reported | 135,660 | 31,447 | 23.2 |
| Total | 5,520,931 | 331,361 | 6.0 |

NH: Non-Hispanic


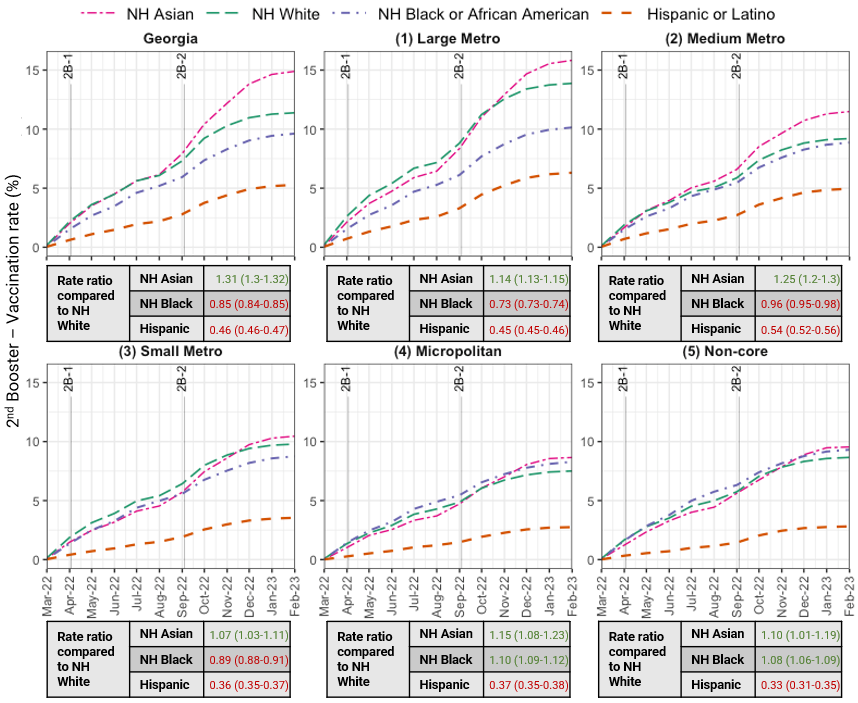


Important dates are labeled as follows: 2^nd^ booster recommendations – 2B-1: Older adults and immunocompromised (April 2022), 2B-2: Updated, bivalent booster for individuals 12+ (September 2022).

NH: Non-Hispanic

**Figure 1**. Monthly cumulative 2^nd^ booster vaccination rate (county of residence not imputed) by county urban-rural classification, stratified by race/ethnicity through February 28, 2023.

Rate ratios (95% confidence intervals) of the vaccination rates of non-Hispanic (NH) Asian, NH Black, and Hispanic adults compared to NH White adults are displayed. The plot shows no imputed vaccination rates.


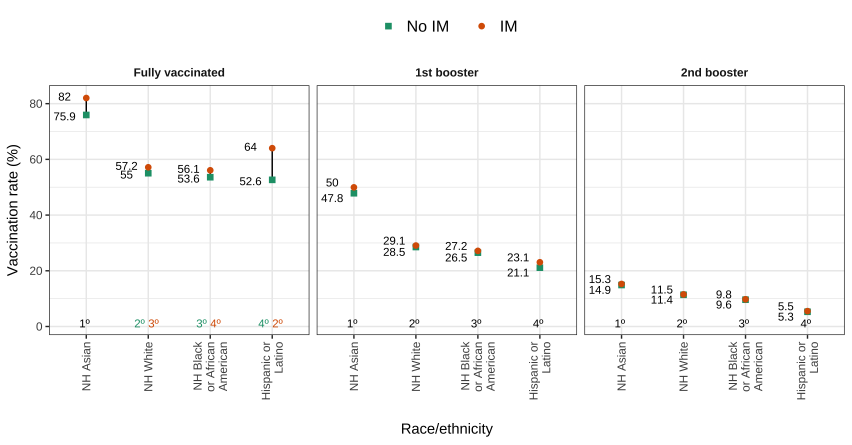


NH: Non-Hispanic

**Figure 2.** Not imputed (No IM) and imputed (IM) cumulative vaccination rates through February 28, 2023, by race/ethnicity in Georgia.

The number at the bottom of the plots indicates the vaccination ranking among the groups.

**Table 4.** Cumulative vaccination rates (county of residence not imputed) and rate ratios (RRs) in Georgia and by county urban-rural classification through February 28, 2023.

| **County Class** | **Race/Ethnicity** | **Fully Vaccinated (%)** | | **1^st^ Booster (%)** | | **2^nd^ Booster (%)** | |
| --- | --- | --- | --- | --- | --- | --- | --- |
|  |  | Crude Rate | Rate Ratio (95% CI) | Crude Rate | Rate Ratio (95% CI) | Crude Rate | Rate Ratio (95% CI) |
| Georgia | NH Asian | 75.94 | 1.38 (1.38-1.38) | 47.84 | 1.68 (1.67-1.68) | 14.89 | 1.31 (1.3-1.32) |
|  | NH Black or African American | 53.59 | 0.97 (0.97-0.98) | 26.51 | 0.93 (0.93-0.93) | 9.62 | 0.85 (0.84-0.85) |
|  | NH White | 55.03 | 1 | 28.53 | 1 | 11.38 | 1 |
|  | Hispanic or Latino | 52.65 | 0.96 (0.95-0.96) | 21.11 | 0.74 (0.74-0.74) | 5.28 | 0.46 (0.46-0.47) |
|  | All races/ethnicities | 63.53 | - | 32.43 | - | 12.15 | - |
| (1) Large Metro | NH Asian | 78.09 | 1.31 (1.31-1.32) | 50.12 | 1.52 (1.51-1.52) | 15.83 | 1.14 (1.13-1.15) |
|  | NH Black or African American | 54.53 | 0.92 (0.92-0.92) | 27.53 | 0.83 (0.83-0.84) | 10.14 | 0.73 (0.73-0.74) |
|  | NH White | 59.47 | 1 | 33.01 | 1 | 13.88 | 1 |
|  | Hispanic or Latino | 57.27 | 0.96 (0.96-0.97) | 24.11 | 0.73 (0.73-0.73) | 6.31 | 0.45 (0.45-0.46) |
|  | All races/ethnicities | 68.51 | - | 36.65 | - | 14.08 | - |
| (2) Medium Metro | NH Asian | 68.73 | 1.34 (1.32-1.35) | 38.73 | 1.57 (1.54-1.59) | 11.49 | 1.25 (1.2-1.3) |
|  | NH Black or African American | 51.71 | 1.01 (1-1.01) | 24.56 | 0.99 (0.99-1) | 8.86 | 0.96 (0.95-0.98) |
|  | NH White | 51.39 | 1 | 24.71 | 1 | 9.20 | 1 |
|  | Hispanic or Latino | 44.55 | 0.87 (0.86-0.88) | 17.89 | 0.72 (0.71-0.74) | 4.96 | 0.54 (0.52-0.56) |
|  | All races/ethnicities | 59.41 | - | 28.57 | - | 10.46 | - |
| (3) Small Metro | NH Asian | 62.79 | 1.2 (1.19-1.21) | 36.10 | 1.41 (1.38-1.43) | 10.46 | 1.07 (1.03-1.11) |
|  | NH Black or African American | 51.89 | 0.99 (0.99-0.99) | 24.83 | 0.97 (0.96-0.97) | 8.75 | 0.89 (0.88-0.91) |
|  | NH White | 52.45 | 1 | 25.69 | 1 | 9.79 | 1 |
|  | Hispanic or Latino | 44.99 | 0.86 (0.85-0.86) | 16.15 | 0.63 (0.62-0.64) | 3.54 | 0.36 (0.35-0.37) |
|  | All races/ethnicities | 58.52 | - | 28.08 | - | 10.17 | - |
| (4) Micropolitan | NH Asian | 63.99 | 1.3 (1.28-1.32) | 35.56 | 1.63 (1.59-1.68) | 8.65 | 1.15 (1.08-1.23) |
|  | NH Black or African American | 51.26 | 1.04 (1.04-1.05) | 24.10 | 1.1 (1.09-1.11) | 8.29 | 1.1 (1.09-1.12) |
|  | NH White | 49.20 | 1 | 21.84 | 1 | 7.51 | 1 |
|  | Hispanic or Latino | 48.85 | 0.99 (0.98-1) | 15.82 | 0.72 (0.71-0.74) | 2.75 | 0.37 (0.35-0.38) |
|  | All races/ethnicities | 54.85 | - | 24.46 | - | 8.19 | - |
| (5) Non-core | NH Asian | 69.64 | 1.44 (1.41-1.46) | 37.09 | 1.6 (1.55-1.65) | 9.54 | 1.1 (1.01-1.19) |
|  | NH Black or African American | 54.21 | 1.12 (1.11-1.12) | 26.49 | 1.14 (1.13-1.15) | 9.31 | 1.08 (1.06-1.09) |
|  | NH White | 48.48 | 1 | 23.22 | 1 | 8.66 | 1 |
|  | Hispanic or Latino | 39.61 | 0.82 (0.81-0.83) | 13.70 | 0.59 (0.57-0.61) | 2.82 | 0.33 (0.31-0.35) |
|  | All races/ethnicities | 54.35 | - | 25.97 | - | 9.40 | - |

NH: Non-Hispanic

Rate ratios (95% confidence intervals) are computed using age-adjusted rates and non-Hispanic (NH) White as the reference population.

**Table 5.** Cumulative crude and age-adjusted COVID-19-related deaths and COVID-19-positive hospitalizations in Georgia and by county urban-rural classification through February 28, 2023.

| **County Class** | **Race/Ethnicity** | **COVID-19-Related Deaths** | | | **COVID-19-Positive Hospitalizations** | | |
| --- | --- | --- | --- | --- | --- | --- | --- |
|  |  | Crude Rate | Age-Adjusted Rate | Rate Ratio (95% CI) | Crude Rate | Age-Adjusted Rate | Rate Ratio (95% CI) |
| Georgia | NH Asian | 181.6 | 264.8 | 0.57 (0.53-0.62) | 598.3 | 743.3 | 0.56 (0.53-0.58) |
|  | NH Black or African American | 511.6 | 630.1 | 1.37 (1.34-1.39) | 2,156.2 | 2,368.6 | 1.77 (1.75-1.79) |
|  | NH White | 565.9 | 460.6 | 1 | 1,518.8 | 1,337.4 | 1 |
|  | Hispanic or Latino | 268.3 | 520.8 | 1.13 (1.07-1.19) | 1,537.5 | 2,036.7 | 1.52 (1.49-1.56) |
|  | All races/ethnicities | 501.9 | 501.9 | - | 1,701.8 | 1,698.9 | - |
| (1) Large Metro | NH Asian | 162.8 | 249.5 | 0.64 (0.58-0.7) | 572.6 | 728.0 | 0.63 (0.6-0.67) |
|  | NH Black or African American | 403.2 | 542.1 | 1.39 (1.35-1.44) | 2,007.5 | 2,256.4 | 1.96 (1.93-1.99) |
|  | NH White | 452.2 | 389.3 | 1 | 1,264.5 | 1,151.0 | 1 |
|  | Hispanic or Latino | 263.3 | 526.7 | 1.35 (1.26-1.45) | 1,583.5 | 2,081.3 | 1.81 (1.76-1.86) |
|  | All races/ethnicities | 394.7 | 435.0 | - | 1,524.70 | 1,581.9 | - |
| (2) Medium Metro | NH Asian | 242.3 | 272.4 | 0.64 (0.46-0.84) | 674.1 | 729.6 | 0.57 (0.45-0.64) |
|  | NH Black or African American | 563.5 | 635.0 | 1.49 (1.40-1.59) | 2,279.5 | 2,447.7 | 1.93 (1.87-2.0) |
|  | NH White | 514.9 | 425.9 | 1 | 1,422.5 | 1,269.7 | 1 |
|  | Hispanic or Latino | 210.9 | 366.4 | 0.86 (0.69-1.04) | 1,023.7 | 1,424.1 | 1.12 (1.02-1.24) |
|  | All races/ethnicities | 507.8 | 489.0 | - | 1,694.6 | 1,669.6 | - |
| (3) Small metro | NH Asian | 254.0 | 349.3 | 0.7 (0.53-0.86) | 697.4 | 858.9 | 0.56 (0.45-0.62) |
|  | NH Black or African American | 649.0 | 748.3 | 1.5 (1.42-1.57) | 2,492.8 | 2,696.1 | 1.76 (1.71-1.81) |
|  | NH White | 623.6 | 499.3 | 1 | 1,746.5 | 1,534.0 | 1 |
|  | Hispanic or Latino | 276.7 | 564.8 | 1.13 (0.99-1.28) | 1,665.4 | 2,314.5 | 1.51 (1.42-1.59) |
|  | All races/ethnicities | 581.0 | 554.7 | - | 1,926.80 | 1,897.4 | - |
| (4) Micropolitan | NH Asian | 295.2 | 343.3 | 0.52 (0.34-0.73) | 726.7 | 795.7 | 0.49 (0.35-0.59) |
|  | NH Black or African American | 821.1 | 914.7 | 1.39 (1.31-1.47) | 2,441.5 | 2,624.1 | 1.61 (1.55-1.66) |
|  | NH White | 832.7 | 659.9 | 1 | 1,882.7 | 1,634.0 | 1 |
|  | Hispanic or Latino | 335.2 | 634.4 | 0.96 (0.81-1.13) | 1,420.5 | 1,900.8 | 1.16 (1.06-1.26) |
|  | All races/ethnicities | 783.0 | 712.0 | - | 2,026.1 | 1,936.5 | - |
| (5) Non-core | NH Asian | 414.2 | 515.0 | 0.93 (0.59-1.29) | 983.8 | 1,081.2 | 0.65 (0.45-0.79) |
|  | NH Black or African American | 807.9 | 796.3 | 1.44 (1.36-1.53) | 2,343.2 | 2,330.2 | 1.4 (1.35-1.45) |
|  | NH White | 785.8 | 552.6 | 1 | 2,076.2 | 1,664.9 | 1 |
|  | Hispanic or Latino | 285.0 | 461.2 | 0.83 (0.66-1.02) | 1,422.5 | 1,800.3 | 1.08 (0.98-1.19) |
|  | All races/ethnicities | 754.0 | 595.7 | - | 2,123.4 | 1,850.4 | - |

NH: Non-Hispanic

Rate ratios (95% confidence intervals) are computed using age-adjusted rates and non-Hispanic (NH) White as the reference population.

**Table 6.** Cumulative age-adjusted COVID-19-related deaths per 100,000 adults by vaccination status in Georgia and by county urban-rural classification through February 28, 2023.

|  | | **NH White** | | **NH Asian** | | **NH Black** | | **Hispanic or Latino** | |
| --- | --- | --- | --- | --- | --- | --- | --- | --- | --- |
|  |  | Age-Adjusted | RR to NH White | Age-Adjusted | RR to NH White  (95% CI) | Age-Adjusted | RR to NH White  (95% CI) | Age-Adjusted | RR to NH White  (95% CI) |
| **Unvaccina-ted or Partially Vaccinated** | Georgia | 664.8 | 1 | 459.6 | 0.69 (0.61 - 0.77) | 889.7 | 1.34 (1.3-1.38) | 649.3 | 0.98 (0.9-1.06) |
|  | (1) Large Metro | 612.0 |  | 510.4 | 0.83 (0.72 - 0.94) | 835.8 | 1.37 (1.3-1.43) | 815.8 | 1.33 (1.2-1.48) |
|  | (2) Medium Metro | 569.9 |  | 231.2 | 0.41 (0.22 - 0.6) | 914.3 | 1.6 (1.47-1.75) | 539.5 | 0.95 (0.7-1.27) |
|  | (3) Small Metro | 739.9 |  | 474.0 | 0.64 (0.43 - 0.89) | 957.1 | 1.29 (1.19-1.4) | 481.9 | 0.65 (0.54-0.79) |
|  | (4) Micropolitan | 889.3 |  | 392.4 | 0.44 (0.22 - 0.7) | 1,213.7 | 1.36 (1.25-1.49) | 608.4 | 0.68 (0.53-0.89) |
|  | (5) Non-core | 668.5 |  | 498.4 | 0.75 (0.37 - 1.28) | 905.5 | 1.35 (1.22-1.5) | 360.9 | 0.54 (0.38-0.76) |
| **Primary Series Only** | Georgia | 231.2 |  | 191.0 | 0.83 (0.62 - 1.06) | 297.6 | 1.29 (1.19-1.38) | 122.6 | 0.53 (0.39-0.69) |
|  | (1) Large Metro | 239.2 |  | 192.2 | 0.8 (0.58 - 1.08) | 271.8 | 1.14 (1.01-1.27) | 116.1 | 0.49 (0.33-0.67) |
|  | (2) Medium Metro | 215.2 |  | 136.9 | 0.64 (0.13 -1.34) | 349.1 | 1.62 (1.29-1.98) | 58.1 | 0.27 (0-0.72) |
|  | (3) Small Metro | 248.1 |  | 188.6 | 0.76 (0.17 - 1.5) | 365.2 | 1.47 (1.19-1.79) | 221.3 | 0.89 (0.44-1.41) |
|  | (4) Micropolitan | 224.0 |  | 331.1 | 1.48 (0.16 - 3.17) | 343.5 | 1.53 (1.19-1.91) | 62.2 | 0.28 (0-0.71) |
|  | (5) Non-core | 213.4 |  | 216.0 | 1.01 (0 - 2.73) | 217.8 | 1.02 (0.76-1.32) | 148.1 | 0.69 (0-1.77) |
| **Primary Series and 1^st^ Booster** | Georgia | 52.6 |  | 29.4 | 0.56 (0.38 - 0.74) | 50.3 | 0.96 (0.86-1.07) | 30.5 | 0.58 (0.4-0.79) |
|  | (1) Large Metro | 48.8 |  | 29.8 | 0.61 (0.42 - 0.82) | 44.8 | 0.92 (0.79-1.06) | 33.0 | 0.68 (0.42-0.96) |
|  | (2) Medium Metro | 60.5 |  | 65.8 | 1.09 (0.33 - 2.14) | 61.5 | 1.02 (0.77-1.33) | 40.8 | 0.67 (0.14-1.49) |
|  | (3) Small Metro | 56.0 |  | - | - | 44.8 | 0.8 (0.58-1.08) | 24.4 | 0.44 (0.08-0.99) |
|  | (4) Micropolitan | 56.2 |  | - | - | 68.8 | 1.23 (0.86-1.67) | - | - |
|  | (5) Non-core | 61.3 |  | - | - | 68.1 | 1.11 (0.71-1.67) | 24.8 | 0.4 (0-1.39) |

NH: Non-Hispanic; RR: Rate Ratio

County of residence was not imputed in the vaccination population counts used in the calculations. Rate ratios (95% confidence intervals) are computed with non-Hispanic (NH) White as the reference population.

**Table 7**. Cumulative age-adjusted COVID-19-positive hospitalizations per 100,000 adults by vaccination status in Georgia and by county urban-rural classification as of February 28, 2023.

|  | | **NH White** | | **NH Asian** | | **NH Black** | | **Hispanic or Latino** | |
| --- | --- | --- | --- | --- | --- | --- | --- | --- | --- |
|  |  | Age-Adjusted | RR to NH White | Age-Adjusted | RR to NH White  (95% CI) | Age-Adjusted | RR to NH White  (95% CI) | Age-Adjusted | RR to NH White  (95% CI) |
| **Unvaccina-ted or Partially Vaccinated** | Georgia | 1,936.5 | 1 | 1,407.7 | 0.73 (0.68 - 0.77) | 3,428.9 | 1.77 (1.74 - 1.8) | 2,244.6 | 1.16 (1.11 - 1.2) |
|  | (1) Large Metro | 1,821.4 |  | 1,608.4 | 0.88 (0.82 - 0.95) | 3,550.3 | 1.95 (1.91 - 2) | 2,708.7 | 1.49 (1.41 - 1.56) |
|  | (2) Medium Metro | 1,765.4 |  | 746.4 | 0.42 (0.31 - 0.55) | 3,468.0 | 1.96 (1.88 - 2.05) | 2,056.4 | 1.16 (1 - 1.34) |
|  | (3) Small Metro | 2,219.2 |  | 1,121.1 | 0.51 (0.4 - 0.62) | 3,498.9 | 1.58 (1.51 - 1.64) | 1,947.8 | 0.88 (0.81 - 0.95) |
|  | (4) Micropolitan | 2,123.5 |  | 1,059.2 | 0.5 (0.33 - 0.67) | 3,252.8 | 1.53 (1.45 - 1.61) | 1,505.9 | 0.71 (0.61 - 0.82) |
|  | (5) Non-core | 2,042.6 |  | 1,445.1 | 0.71 (0.43 - 1) | 2,784.7 | 1.36 (1.29 - 1.44) | 1,282.4 | 0.63 (0.52 - 0.74) |
| **Primary Series Only** | Georgia | 721.4 |  | 445.1 | 0.62 (0.52 - 0.72) | 1,278.3 | 1.77 (1.71 - 1.84) | 543.7 | 0.75 (0.67 - 0.84) |
|  | (1) Large Metro | 698.6 |  | 442.2 | 0.63 (0.53 - 0.75) | 1,299.5 | 1.86 (1.77 - 1.95) | 535.3 | 0.77 (0.67 - 0.88) |
|  | (2) Medium Metro | 705.4 |  | 587.8 | 0.83 (0.48 - 1.24) | 1,481.0 | 2.1 (1.9 - 2.31) | 527.6 | 0.75 (0.44 - 1.07) |
|  | (3) Small Metro | 754.4 |  | 350.8 | 0.46 (0.23 - 0.78) | 1,303.6 | 1.73 (1.57 - 1.89) | 702.7 | 0.93 (0.7 - 1.17) |
|  | (4) Micropolitan | 687.1 |  | 420.0 | 0.61 (0.15 - 1.19) | 1,057.0 | 1.54 (1.35 - 1.72) | 366.4 | 0.53 (0.25 - 0.83) |
|  | (5) Non-core | 827.6 |  | 442.7 | 0.53 (0.07 - 1.19) | 968.4 | 1.17 (1.03 - 1.33) | 499.3 | 0.6 (0.3 - 0.99) |
| **Primary Series and 1^st^ Booster** | Georgia | 251.3 |  | 99.9 | 0.4 (0.33 - 0.47) | 400.6 | 1.59 (1.52 - 1.67) | 181.9 | 0.72 (0.63 - 0.82) |
|  | (1) Large Metro | 229.6 |  | 93.3 | 0.41 (0.33 - 0.49) | 380.7 | 1.66 (1.55 - 1.76) | 154.2 | 0.67 (0.55 - 0.79) |
|  | (2) Medium Metro | 274.4 |  | 131.5 | 0.48 (0.22 - 0.77) | 489.1 | 1.78 (1.57 - 2.04) | 176.3 | 0.64 (0.33 - 0.99) |
|  | (3) Small Metro | 283.1 |  | 145.1 | 0.51 (0.25 - 0.84) | 444.7 | 1.57 (1.37 - 1.79) | 317.6 | 1.12 (0.84 - 1.47) |
|  | (4) Micropolitan | 266.1 |  | 160.4 | 0.6 (0.11 - 1.26) | 409.0 | 1.54 (1.27 - 1.85) | 153.9 | 0.58 (0.24 - 0.96) |
|  | (5) Non-core | 341.0 |  | 81.5 | 0.24 (0 - 0.62) | 349.9 | 1.03 (0.85 - 1.22) | 223.8 | 0.66 (0.3 - 1.12) |

NH: Non-Hispanic; RR: Rate Ratio

County of residence was not imputed in the vaccination population counts used in the calculations. Rate ratios (95% confidence intervals) are computed with non-Hispanic (NH) White as the reference population.

**Appendix B. County-level analysis**

A county-level analysis was performed to understand county-to-county vaccination, death, and hospitalization rate variations. Rates were calculated individually by race/ethnic group and the mean and standard deviation of the counties’ rates were calculated by county urban-rural class. Counties in which the death and hospitalization counts per racial/ethnic group were less than 5 were removed. Additionally, extreme outliers (< Q1 (first quartile) – 4*IQR (interquartile range) or > Q3 + 4*IQR) were removed. 95% confidence intervals were computed using one-sample z-tests.

Due to small counts in deaths and hospitalizations (< 5), 19 and 9 counties were excluded in the death and hospitalization analysis, respectively, in the non-Hispanic (NH) Black and NH White comparison, while 103 and 9 counties were excluded in the Hispanic and NH White comparison. Additional counties were removed since they were extreme outliers (3 counties in the NH Black and NH White comparison and 8 counties in the Hispanic and NH White comparison). NH Black adults had higher vaccination coverage than NH White adults in the majority of counties (63.5 to 70.3% of the counties, depending on the vaccine dose) (Figure B1). However, the vaccination of NH White adults was higher than the vaccination of NH Black adults in the largest counties. Most of the counties had higher NH Black age-adjusted death (86.3% of the counties) and hospitalization (95.3% of the counties) rates than their NH White counterparts (Figure B1). NH White adults had higher vaccination coverage than Hispanic adults in the majority of counties, especially for the booster doses (67.3 to 96.2% of the counties, depending on the vaccine dose) and lower age-adjusted death and hospitalization rates (62.7 and 66% of the counties, respectively) (Figure B2). Table 8 shows the average rate ratios and confidence intervals per county class. Due to the data censoring, the number of counties included is limited, especially in the most rural county classes and for the NH Asian and Hispanic groups.

**Figure B1.** Vaccination (not imputed) and age-adjusted death and hospitalization rates for non-Hispanic (NH) Black and NH White adults by county.


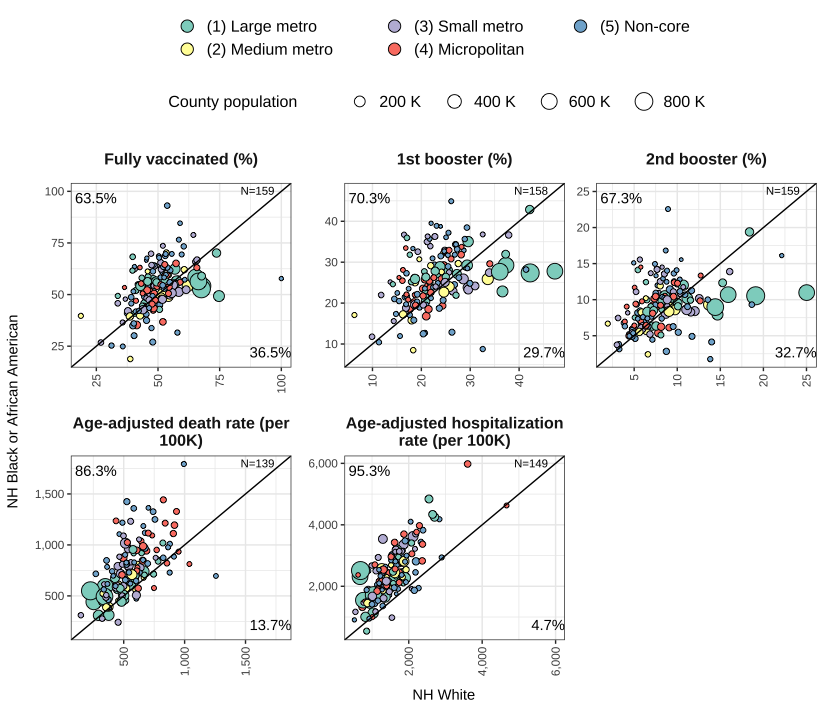


NH: Non-Hispanic

The text in the plot shows the percentage of counties in which the NH Black rate was higher than the NH White rate (above the line) and vice versa. Counties were removed if their vaccination or outcome counts were less than 5 per racial/ethnic group or if they were extreme outliers (< Q1 – 4*IQR or > Q3 + 4*IQR).

**Figure B2**. Vaccination (not imputed) and age-adjusted death and hospitalization rates for Hispanic and non-Hispanic (NH) White adults by county.


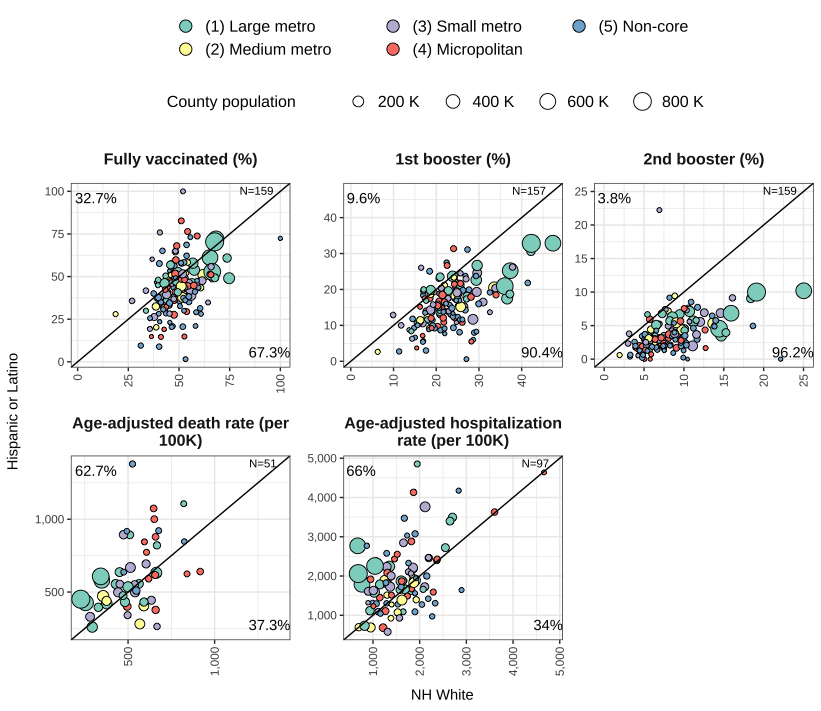


NH: Non-Hispanic

The text in the plot shows the percentage of counties in which the Hispanic rate was higher than the NH White rate (above the line) and vice versa. Counties were removed if their vaccination or outcome counts were less than 5 per racial/ethnic group or if they were extreme outliers (< Q1 – 4*IQR or > Q3 + 4*IQR).

| County Class | N |  | Fully Vaccinated | | 1^st^ Booster | | 2^nd^ Booster | | Age-Adjusted Deaths | | | Age-Adjusted Hospitalizations | | |
| --- | --- | --- | --- | --- | --- | --- | --- | --- | --- | --- | --- | --- | --- | --- |
|  |  | **N**^a^ | **RR - Mean (SD)** | **95% CI** | **RR - Mean (SD)** | **95% CI** | **RR - Mean (SD)** | **95% CI** | **N** | **RR - Mean (SD)** | **95% CI** | **N** | **RR - Mean (SD)** | **95% CI** |
| Georgia | NH Asian | 159 | 1.3 (0.4) | (1.2-1.3) | 1.6 (0.7) | (1.5-1.7) | 1.2 (0.8) | (1-1.3) | 23 | 0.8 (0.3) | (0.7-0.9) | 36 | 0.7 (0.2) | (0.6-0.7) |
|  | NH Black or African American |  | 1.1 (0.2) | (1.1-1.1) | 1.2 (0.4) | (1.1-1.2) | 1.2 (0.5) | (1.1-1.3) | 140 | 1.4 (0.5) | (1.4-1.5) | 150 | 1.7 (0.8) | (1.6-1.8) |
|  | Hispanic or Latino |  | 0.9 (0.3) | (0.8-0.9) | 0.7 (0.4) | (0.7-0.8) | 0.5 (0.3) | (0.4-0.5) | 56 | 1.4 (1) | (1.2-1.7) | 98 | 1.3 (0.7) | (1.2-1.5) |
| (1) Large Metro | NH Asian | 28 | 1.3 (0.3) | (1.3-1.4) | 1.6 (0.5) | (1.4-1.8) | 1.2 (0.3) | (1.1-1.3) | 11 | 0.8 (0.2) | (0.6-0.9) | 17 | 0.7 (0.2) | (0.6-0.8) |
|  | NH Black or African American |  | 1.1 (0.2) | (1-1.2) | 1.1 (0.4) | (1-1.2) | 1.1 (0.4) | (0.9-1.2) | 26 | 1.3 (0.3) | (1.2-1.4) | 27 | 1.6 (0.6) | (1.4-1.9) |
|  | Hispanic or Latino |  | 1 (0.1) | (0.9-1) | 0.8 (0.2) | (0.7-0.8) | 0.6 (0.2) | (0.5-0.6) | 20 | 1.5 (1) | (1-1.9) | 23 | 1.5 (0.8) | (1.2-1.8) |
| (2) Medium Metro | NH Asian | 14 | 1.2 (0.3) | (1.1-1.4) | 1.4 (0.4) | (1.2-1.6) | 1.1 (0.4) | (0.9-1.3) | 4 | 0.6 (0.1) | (0.5-0.7) | 4 | 0.6 (0.1) | (0.5-0.7) |
|  | NH Black or African American |  | 1.1 (0.4) | (0.9-1.3) | 1.2 (0.5) | (0.9-1.4) | 1.2 (0.7) | (0.8-1.6) | 12 | 1.2 (0.2) | (1.1-1.3) | 13 | 1.6 (0.3) | (1.5-1.8) |
|  | Hispanic or Latino |  | 0.9 (0.3) | (0.8-1.1) | 0.8 (0.2) | (0.7-0.9) | 0.6 (0.2) | (0.5-0.7) | 4 | 0.9 (0.4) | (0.5-1.3) | 11 | 0.9 (0.2) | (0.8-1.1) |
| (3) Small Metro | NH Asian | 28 | 1.2 (0.3) | (1.1-1.3) | 1.5 (0.8) | (1.2-1.8) | 1.1 (1.2) | (0.7-1.6) | 6 | 0.9 (0.4) | (0.5-1.2) | 12 | 0.6 (0.2) | (0.5-0.8) |
|  | NH Black or African American |  | 1.1 (0.2) | (1-1.2) | 1.2 (0.4) | (1.1-1.4) | 1.3 (0.6) | (1.1-1.5) | 25 | 1.5 (0.4) | (1.3-1.7) | 26 | 1.7 (0.5) | (1.5-1.9) |
|  | Hispanic or Latino |  | 1 (0.4) | (0.8-1.1) | 0.9 (0.8) | (0.6-1.2) | 0.6 (0.6) | (0.4-0.8) | 13 | 1.5 (1.2) | (0.9-2.1) | 16 | 1.3 (0.4) | (1.1-1.5) |
| (4) Micropoli-tan | NH Asian | 31 | 1.3 (0.4) | (1.2-1.5) | 1.8 (0.8) | (1.6-2.1) | 1.4 (0.9) | (1.1-1.7) | 1 | 1 | NA | 2 | 0.9 (0.2) | NA |
|  | NH Black or African American |  | 1.1 (0.2) | (1-1.1) | 1.2 (0.3) | (1.1-1.3) | 1.3 (0.4) | (1.1-1.4) | 28 | 1.4 (0.4) | (1.2-1.5) | 31 | 1.7 (0.5) | (1.5-1.9) |
|  | Hispanic or Latino |  | 0.9 (0.3) | (0.8-1) | 0.7 (0.3) | (0.6-0.8) | 0.4 (0.2) | (0.3-0.5) | 12 | 1.2 (0.6) | (0.9-1.5) | 20 | 1.3 (0.5) | (1.1-1.5) |
| (5) Non-core | NH Asian | 58 | 1.2 (0.4) | (1.1-1.3) | 1.5 (0.6) | (1.3-1.6) | 1.1 (0.8) | (0.9-1.3) | 1 | 1.4 | NA | 1 | 0.8 | NA |
|  | NH Black or African American |  | 1.1 (0.2) | (1-1.2) | 1.2 (0.3) | (1.1-1.2) | 1.2 (0.5) | (1.1-1.3) | 49 | 1.6 (0.7) | (1.4-1.8) | 53 | 1.7 (1.1) | (1.4-2) |
|  | Hispanic or Latino |  | 0.8 (0.3) | (0.7-0.9) | 0.6 (0.3) | (0.5-0.7) | 0.4 (0.2) | (0.3-0.4) | 7 | 2 (1.3) | (1-3) | 28 | 1.4 (1) | (1-1.7) |

**Table B1**. Mean vaccination, age-adjusted death, and hospitalization rate ratios across counties, grouped by county urban-rural classification.

^a^ Sample size for fully vaccinated, 1st booster, and 2nd booster.

NH: Non-Hispanic; RR: Rate Ratio; SD: Standard Deviation; CI: Confidence Interval

Counties where death and hospitalization counts were less than 5 per racial/ethnic group were not included.

**References**

1. U.S. Census Bureau. 2022: American Community Survey (ACS) Demographic and Housing Estimates 5-Year Estimates, Table ID DP05. 2022.

2. OASIS (Online Analytical Statistical Information System) Web Query - Population Statistics [Internet]. Available from: <https://oasis.state.ga.us/oasis/webquery/qryPopulation.aspx>.

3. Schluter D. Biology 548b: Quantitative Methods in Ecology and Evolution - Lecture 10: Bootstrap The University of British Columbia. 2012 [Available from: <https://www.zoology.ubc.ca/biol548/lecturepdf/10.Bootstrap.pdf>.
